# P-530. Mortality among U.S. Children <18 Years Old Hospitalized with Laboratory-Confirmed COVID-19 Infection, 12 States, March 2020–September 2023

**DOI:** 10.1093/ofid/ofaf695.745

**Published:** 2026-01-11

**Authors:** Jiana baker, Christopher A Taylor, Jennifer Milucky, Kadam Patel, Monica E P Patton, Shua J Chai, Julie Plano, Kyle P P Openo, Patricia A Ryan, Elizabeth Reeg, Paige D’Heilly, Dominic Solhtalab, Adam Rowe, Sophrena Bushey, Melissa Sutton, Kristen Olsen, Fiona P Havers, Dennis Wang

**Affiliations:** Florida International University, Miami, Florida; Centers for Disease Control and Prevention, Atlanta, Georgia; CDC, Atlanta, Georgia; Centers for Disease Control and Prevention, Atlanta, Georgia; Centers for Disease Control and Prevention, Atlanta, Georgia; California Emerging Infections Program, Oakland, CA; Division of State and Local Readiness, CDC, Atlanta, Atlanta, Georgia; Connecticut Emerging Infections Program, Yale School of Public Health, New Haven, Connecticut; Emory University School of Medicine, Atlanta, Georgia; Maryland Department of Health, Baltimore, Maryland; Michigan Department of Health and Human Services, Lansing, Michigan; Minnesota Department of Health, St. Paul, Minnesota; New Mexico Emerging Infections Program, Albuquerque, New Mexico; New York State Department of Health, Albany, New York; University of Rochester School of Medicine and Dentistry, Emerging Infections Program, Rochester, New York; Public Health Division, Oregon Health Authority, Portland, Oregon; Salt Lake County Health Department, Salt Lake City, Utah; Centers for Disease Control and Prevention, Atlanta, Georgia; National Center for Immunization and Respiratory Diseases, Centers for Disease Control and Preventio, Atlanta, Georgia

## Abstract

**Background:**

Pediatric COVID-19 hospitalizations have been well-described, but pediatric COVID-19-associated mortality data are limited.

**Figure:**
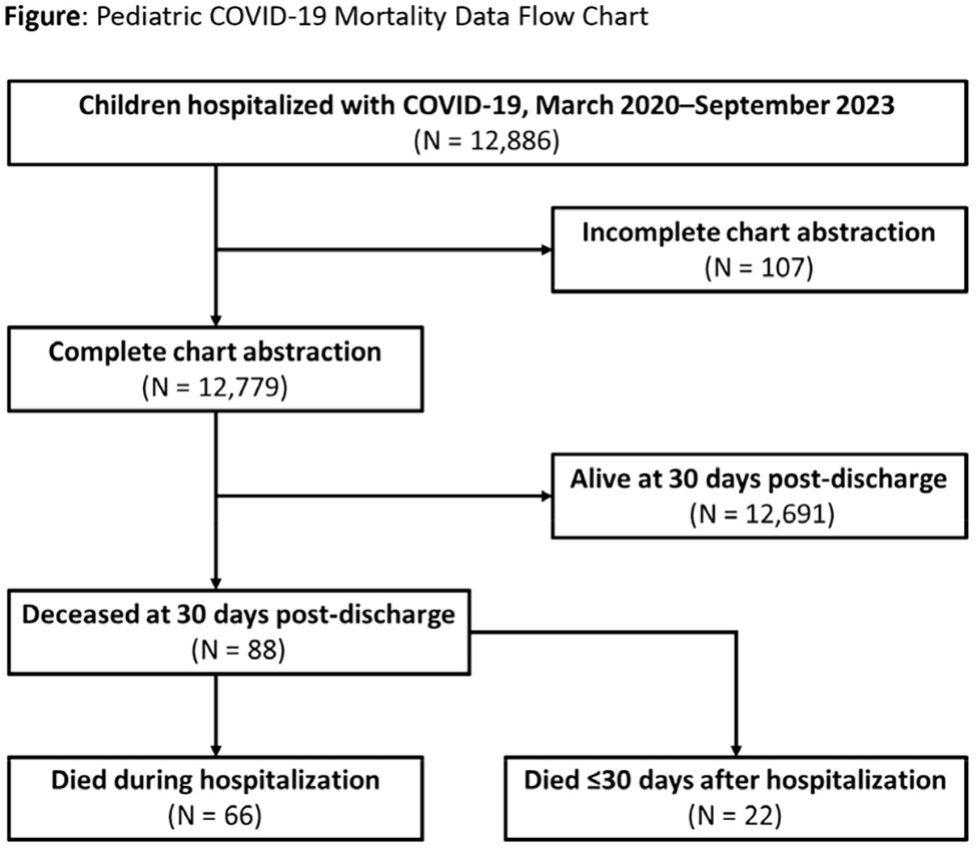

**Figure:**
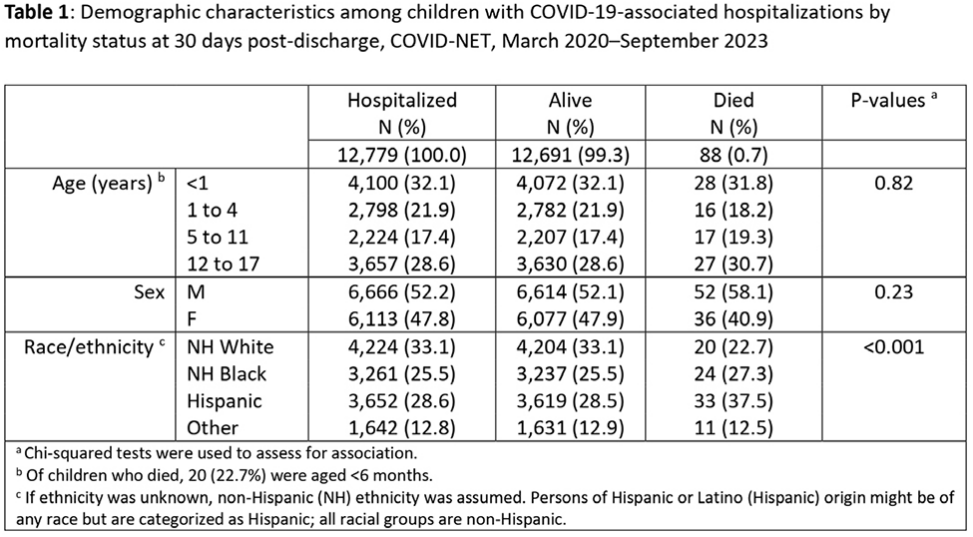

**Methods:**

COVID-19-Associated Hospitalization Surveillance Network (COVID-NET) conducts population-based surveillance for laboratory-confirmed COVID-19 hospitalizations in 12 states. After excluding trauma-related admissions, medical charts among children aged < 18 years hospitalized during March 2020–September 2023 were abstracted. Children who died in-hospital or within 30 days of discharge were identified by matching cases with death certificates to describe pediatric COVID-19-associated mortality.

**Figure:**
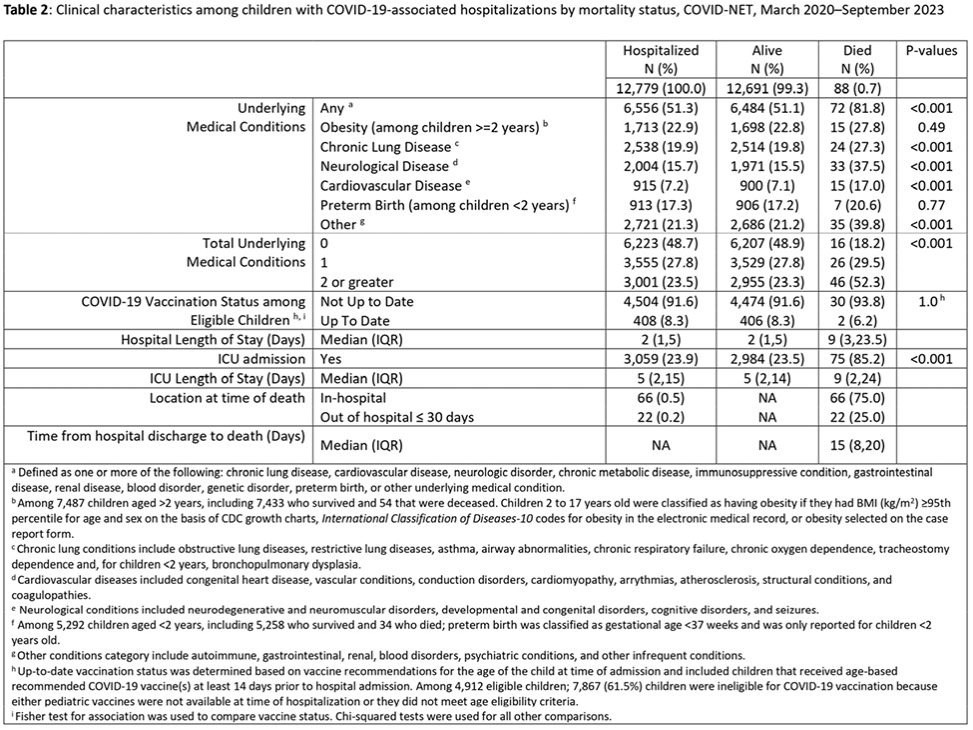

**Results:**

Among 12,779 hospitalized children with complete chart abstraction, 88 (0.7%) died, including 66 (75.0%) in-hospital deaths and 22 (25.0%) deaths that occurred ≤30 days post-discharge (Figure). Thirty-two percent of children who died were < 1 year old and 23% were < 6 months old. Comparing children who died and those who survived, there were no significant differences in age or sex. A higher proportion of children who died than those who survived were Hispanic (37.5% vs 28.5%), while a lower proportion of children who died were non-Hispanic White (22.7% vs 33.1%) (p< 0.001 for overall racial differences) (Table 1). Compared with children who survived, children who died were more likely to have ≥1 (81.8% vs 51.1%) or ≥2 (52.3% vs 23.3%) underlying medical conditions, including neurological (37.5% vs 15.5%), chronic lung (27.3% vs 19.8%), and cardiovascular diseases (17.0% vs 7.0%) (p< 0.001 for all). Among COVID-19 vaccine-eligible children, 93.8% of those who died and 91.6% of those who survived were not up-to-date with COVID-19 vaccine recommendations (Table 2).

**Conclusion:**

While COVID-19-associated pediatric hospitalizations resulting in death were rare, most were among children with comorbidities and COVID-19 vaccine-eligible children who were not up-to-date with vaccination, indicating missed opportunities to prevent severe illness and death through COVID-19 vaccination. One-quarter of deaths occurred within 30 days of discharge, highlighting the need for post-hospitalization surveillance for pediatric COVID-19 mortality estimates.

**Disclosures:**

Melissa Sutton, MD, MPH, Centers for Disease Control and Prevention Emerging Infections Program: Grant/Research Support

